# Improved chemical and isotopic labeling of biomembranes in *Bacillus subtilis* by leveraging CRISPRi inhibition of beta-ketoacyl-ACP synthase (*fabF*)

**DOI:** 10.3389/fmolb.2022.1011981

**Published:** 2022-10-21

**Authors:** Jonathan D. Nickels, Kyle S. Bonifer, Rachel R. Tindall, Ahmad Yahya, Luoxi Tan, Changwoo Do, Brian H. Davison, James G. Elkins

**Affiliations:** ^1^ Department of Chemical and Environmental Engineering, University of Cincinnati, Cincinnati, OH, United States; ^2^ Biosciences Division, Oak Ridge National Laboratory, Oak Ridge, TN, United States; ^3^ Neutron Sciences Division, Oak Ridge National Laboratory, Oak Ridge, TN, United States

**Keywords:** *Bacillus subtilis*, cell membrane, CRISPRi, fatty acid, neutron scattering, SANS, isotopic labelling

## Abstract

Assessing the structure of living microbial cell membranes is a challenging analytical goal. The cell membrane is defined by its transverse structure, an approximately 5 nm-thick selectively permeable bilayer that serves many important cellular functions. Compositionally complex, dynamic, and organized in both the transverse and lateral dimensions, understanding the cell membrane structure—and the role that structure plays in cellular function, communication, and environmental sensing is an active scientific effort. Previously, we have devised a novel isotopic labeling approach for membrane lipids to enable direct *in vivo* structural studies of the cell membrane in the Gram-positive bacterium, *Bacillus subtilis*, using small-angle neutron scattering. This was accomplished through a genetic inhibition of fatty acid (FA) degradation (Δ*fadN*) and a chemical inhibition of FA biosynthesis using cerulenin, an irreversible inhibitor of type II fatty acid synthases. Here, we improve upon the previous system by introducing a dCas9/sgRNA-*fabF* complex that blocks transcription of the essential *fabF* gene when under xylose induction. This leads to greater sensitivity to cerulenin in the mutant strain (JEBS102) and more robust cell growth when supplementary FAs are introduced to the culture medium. A subtle change in FA uptake is noted when compared to the prior labeling strategy. This is seen in the gas chromatography/mass spectrometry (GC/MS) data as a higher ratio of *n*16:0 to *a*15:0, and manifests in an apparent increase in the membrane thickness determined *via* neutron scattering. This represents an improved method of isotopic labeling for the cell membrane of *Bacillus subtilis;* enabling improved investigations of cellular uptake and utilization of FAs, cell membrane structure and organization as a phenotypic response to metabolic and environmental changes.

## Introduction

The cell membrane is a complex and multifunctional structure composed of a diverse set of lipids and proteins. It participates in influencing cell morphology, cellular signaling, regulates metabolic input and output, provides the barrier required to maintain a membrane potential, and forms the fluid platform for all the membrane-associated and transmembrane proteins involved in these tasks. However, measuring the details of the cell membrane structure at the nanoscale in living cells is a significant analytical challenge. *In vitro* approaches on fixed cells or cell derived lipid membranes have leveraged a range of methods including electron microscopy (Henderson and Unwin, 1975), NMR (Henderson et al., 1974; Seelig and Seelig, 1974), X-ray scattering (Engelman, 1970) and neutron scattering (Zaccai et al., 1975; Jacrot, 1976; Büldt et al., 1978), along with light microscopy (Evans and La Celle, 1975), fluorescence (Derzko and Jacobson, 1980) and other methods (Seelig and Seelig, 1980; Marsh, 1981). Yet, *in vivo* measurements of cell membrane structure are more limited, primarily focusing on fluorescence methods (Eggeling et al., 2009).

Recently, progress has been made in measuring the structure and organization of bacterial cell membranes by combining isotopic labeling with small-angle neutron scattering (SANS) (Nickels et al., 2017b). SANS is an excellent method when applied to studying the structure of lipid bilayers in general due to its sensitivity to the neutron scattering length density (SLD), a property which varies by element and isotope. In this method, light elements common to soft materials, specifically hydrogen, contribute more to the SLD profile of the bilayer compared to X-rays which scatter from electron density. The specific sensitivity of neutrons to the isotopes of hydrogen enables experiments which replace natural abundance hydrogen, (99.985% 1H), with its isotope 2H, or deuterium, in the bilayer or solvent. This can significantly alter the SLD profile without changing the chemistry or structure, enabling a suite of techniques known as contrast matching. The scattered intensity is proportional to the square of the difference in SLD between an object and the surrounding material(s), by matching the SLD of two materials in the sample one can eliminate their scattering, leaving only the scattering from other targeted components of interest. This has been used to effectively study model lipid membranes in which this kind of strict isotopic control can be achieved (Büldt et al., 1978; Nickels et al., 2015), but the complexity of living systems requires a more challenging experimental approach.

With contributions to observed neutrons scattering from the solvent and all biomolecules comprising the cell, isolating the scattering from the cell membrane requires extensive biodeuteration efforts. Nickels et al. (2017b) achieved this *via* three main strategies, the first being cultivation of the cells in partially deuterated solvent conditions to control the background deuterium content of the cellular biomolecules. Next, FA synthesis was blocked chemically using cerulenin, an irreversible inhibitor of the essential gene *fabF* encoding β-ketoacyl-ACP synthase (Wille et al., 1975; Price et al., 2001). Growth was rescued by introducing exogenous FAs which are incorporated into cellular lipids. Finally, the potential catabolism of the exogenous FAs was prevented by using a Δ*fadN* (aka yusL) strain of *B. subtilis*, eliminating a critical enzyme in β-oxidation, FadN or enoyl-CoA hydratase (Matsuoka et al., 2007). A combination of two FAs was used to rescue cellular growth in the presence of cerulenin—palmitic acid (normal-hexadecanoic acid, (*n*16:0)) and 12-methyltetradecanoic acid (anteiso-pentadecanoic acid, (*a*15:0)). These two FAs provide a minimal set of high-melting and low-melting FAs (Wille et al., 1975; Nickels et al., 2017b), thought to be sufficient for regulation of membrane fluidity. This combination was able to both rescue cell growth and provide defined neutron contrast, enabling direct observations of the cell membrane thickness and laterally heterogeneous distribution of FAs in the cell membrane using neutrons (Nickels et al., 2017b).

This reliance on cerulenin for *fabF* inhibition is not ideal: the compound is sensitive to temperature and oxidation, cells can develop spontaneous resistance mutations, and its effectiveness is variable not only across suppliers but also across lots from the same supplier (Schujman et al., 2001). Additionally, the cellular response to cerulenin is to greatly increase in the expression *fabF*, which further exacerbates issues with its use as in inhibitor (Schujman et al., 2001; Nickels et al., 2020). Leveraging CRISPR interference (CRISPRi) (Qi et al., 2013; Peters et al., 2016) offers an advance from the earlier approach by effectively placing *fabF* inhibition under xylose repression in a *B. subtilis fadN* mutant background. The resulting strain (JEBS102) reduces the use of cerulenin for *fabF* inhibition while still providing for deuteration and subsequent FA supplementation, as we show *via* mass spectrometry and neutron scattering experiments. By regulating the F*abF* enzyme at the transcription level rather than the post-translational level, a wider range of experiments on membrane composition, physical properties, regulation, and structure of the *B. subtilis* cell become feasible.

## Materials and methods

### Strain construction

All strains and plasmids were obtained from the Bacillus Genetic Stock Center at The Ohio State University, Columbus, Ohio. Bacillus strains were routinely cultivated and maintained using either Luria- Bertani (LB) broth or LB agar plates solidified with 1.5% (*w/v*) Bacto Agar (Difco) or Tryptose Blood Agar Base (with yeast extract) plates (HiMedia Laboratories, Mumbai, India). When required for selection, the following antibiotics were added: erythromycin (Em, 1 mg/L), chloramphenicol (Cm, 5 mg/L), and spectinomycin (Sp, 100 mg/L). Total genomic DNA isolations were performed using a Quick-DNA Fungal/Bacterial Microprep Kit (Zymo Research, Irvine, CA). Plasmid DNA preparations were performed using a Qiagen Plasmid Midi Kit (Qiagen, Germantown, MD). Bacillus DNA transformations were performed according to the protocol developed by Zahler and revised by Yasbin *et al.* (Yasbin et al., 1975). Bacterial strains and PCR primers used for confirmation are provided in Supplementary Tables S1, S2. The *yusL* (*fadN*) deletion strain, BKE32840 (*trpC2*, Δ*fadN*::*erm*) was transformed with plasmid pDR244, which contains a temperature sensitive replicon, to excise the Em resistance cassette as previously described (Koo et al., 2017). Several Em sensitive/Sp resistant colonies were grown overnight in LB broth at 42°C to cure the plasmid. Dilutions were plated to obtain colonies which were patched and screened for Sp sensitivity. Removal of the Em resistance cassette was verified using PCR (HotStar, Qiagen). A confirmed isolate (BKE32840 Δ*fadN* Δ*erm*; designated JEBS100) was transformed with total genomic DNA prepared from 1A1278 (Em^r^ *trpC2 lacA*::P_*xyl*_ *-dcas9*; Peters et al., 2016) and initially screened for Em^r^ colonies which were verified *via* PCR to contain the *P*_*xyl*_ *-dcas9* within the *lacA* locus. This strain (designated JEBS101) was subsequently transformed with total genomic DNA isolated from BEC11340 (Cm^r^ Em^r^ *trpC2 lacA*::P_*xyl*_-dcas9 *amyE*::P_*veg*_-sgRNA (*fabF*); Peters et al., 2016) and screened for Cm^r^ colonies. The final strain, JEBS102 (Em^r^ Cm^r^ *trpC2* Δ*fadN lacA*::P_*xyl*_ *-dcas9 amyE*::P_*veg*_ *-*sgRNA (*fabF*)), was verified *via* PCR to contain the sgRNA construct targeting *fabF* within the *amyE* locus.

### Growth conditions

To examine potential differences in cerulenin sensitivity between JEBS102 and the original BKE32840 strain, 10 ml starter cultures were incubated for 16 h at 37°C with shaking at 250 rpm in M9 minimal medium with 2% (*w/v*) glucose (M9-Gluc) supplemented with 5 mM L-tryptophan and appropriate antibiotics for each strain. For high-throughput growth curves, 96-well plates were prepared with 95 μL of M9-Gluc supplemented as above plus 1% (*w/v*) D-xylose added to each well. Cerulenin was also added at 0, 5, 10, and 20 μg/ml from a fresh 10 mg/ml stock prepared in 100% ethanol. Each condition was established in triplicate. The plates were inoculated with 5 μL of starter culture per well and growth was recorded as optical density at 600 nm using an Epoch 2 microplate spectrophotometer (BioTek/Agilent, Santa Clara, CA) with readings taken every 10 min. The plates were incubated at 37°C with continuous shaking for 48 h. For FA-fed conditions, M9-Gluc was supplemental with FAs to a final concentration of 8 mg/L each of *a*15:0 and *n*16:0 from 25 mg/ml stock solutions dissolved in 100% ethanol, along with 5 g/L of FA-free BSA (Sigma Aldrich, St. Louis, MO) as a carrier to aid bioavailability. Cerulenin (10 mg/ml in ethanol stock solution) was prepared fresh and added immediately prior to inoculation at a final concentration of 20 μg/ml. For dCas9 induction, D-xylose was added to a final concentration of 1% (*w/v*). Growth curves were generated in 96-well plates with the conditions described above with OD_600_ measurements recorded every 15 min. The raw output data was analyzed and plotted using Prism9 (GraphPad Software, San Diego, CA).

For analysis of cell membranes using GC/MS and SANS, cells of *B. subtilis* JEBS102 were serially adapted to grow in M9-Gluc with 5 mM L-tryptophan prepared using 90% (*v/v*) D_2_O as previously described (Nickels et al., 2017b). Cultures supplied with exogenous FAs were established as described above. Cells were harvested in 10 ml aliquots from 50 ml cultures reaching an OD_600_ of 0.5–0.7 which were incubated at 37°C for 16–24 h with shaking at 250 rpm. The cells were pelleted by centrifugation at 6000 *g* for 15 min at 4°C then washed 3 times with 3 ml of phosphate buffered saline (PBS: 10 mM Na_2_HPO_4_, 1.8 mM KH_2_PO_4_, 137 mM NaCl, and 2.7 mM KCl, pH 7.2), prepared with D_2_O at 85% (*v/v*). The washed pellets were resuspended in 500 μL of PBS (pH 6.8) prepared with 85% D_2_O and supplemented with 0.1% (*w/v*) glucose, 10 mM MgSO_4_, and 50 μg/ml cerulenin. These additions preserve cell viability and prevent lysis during long collection times during SANS measurements (Nickels et al., 2017b).

### Gas chromatography/mass spectrometry

GC/MS analysis was performed using an Agilent 5890A gas chromatograph with a 5975C mass-sensitive detector operating in electron-impact mode (Agilent Technologies, Santa Clara, CA). The instrument was equipped with an HP-5ms capillary column (30 m long, 0.25 mm outside diameter, and 0.25 μm coating thickness) using helium at 1 ml/min as the carrier gas. Samples of 1 μL were introduced using splitless injection at an inlet temperature of 270°C. Peak assignment, integration, and mass spectral analysis were performed using the instrument’s ChemStation Enhanced Data Analysis software and the NIST mass spectral database.

### Fatty acid methyl ester preparation

Sample preparation for GC/MS followed our prior work (Nickels et al., 2017b) which used a modification (Lewis et al., 2000) of the method of Bligh and Dyer (Bligh and Dyer, 1959). Briefly, this begins by pelleting cellular materials by centrifugation at 6000 × g for 15 min, followed by three washes in 1% (*w/v*) NaCl. Samples are then lyophilized in 10 ml glass test tubes with Teflon-faced screw caps, to each of which was sequentially added 0.5 ml of chloroform, 1 ml of methanol, and 0.4 ml of water, with vigorous agitation at each stage. This mixture forms a single phase and was left to stand for 18 h at room temperature with occasional agitation. After 18 h, phase separation was induced by the addition of 0.5 ml of chloroform and 0.5 ml water. The lipids were recovered from the lower chloroform phase into a new 10 ml glass test tube and solvents removed by evaporation under an argon stream. FAMEs were then produced *via* acidic methanolysis from dried lipid films (Ichihara and Fukubayashi, 2010). Then 1 ml of concentrated HCl/methanol (10% *v/v*) was added, and the test tube purged with argon, sealed, and heated to 85°C for 2 h. After cooling, 1 ml of water and 1 ml of hexane were added, and the contents vortexed. After phase separation, a portion (approximately 700 μL) of the upper phase was sampled for GC/MS analysis.

### Small-angle neutron scattering

SANS data were collected on the Extended Q-range Small Angle Neutron Spectrometer (EQ-SANS) (Zhao et al., 2010) at the at the Spallation Neutron Source located at Oak Ridge National Laboratory. At EQ-SANS, the cell membrane structure was collected in 60 Hz mode with two instrumental configurations: 1.3-m sample-to-detector distance with 4 ± 7 Å neutrons (*q* = 0.05–0.4 Å^−1^) and 4.0 m sample to detector distance with 10 ± 13.4 Å neutrons (*q* = 0.009–0.07 Å^−1^), yielding a total *q*-range from approximately 0.009–0.4 Å^−1^. Two-dimensional scattering data was reduced using the Mantid software (Arnold et al., 2014) and normalized to a porous silica standard to establish an absolute scale, and corrected for pixel sensitivity, dark current, and sample transmission. Background scattering was subtracted from the 1D intensity versus scattering wave vector, *q*. The residual background was recorded using unlabeled cells at the same solvent condition. The self-consistent slab model (Tan et al., 2021) was used to fit the one-dimensional intensity versus q data using the SASview suite (Pencer et al., 2005). This represents the lipid structure as a lamellar form factor representing the hydrocarbon core of the bilayer, with two identical head group regions on either side, within a continuous region representing the scattering length density of the surrounding cellular environment. This experiment matched the headgroup region and solvent, so only the acyl core presented strong contrast. Collection times did not exceed 4 h, at which point cells were determined to be better than 90% viable, as shown in prior work (Nickels et al., 2017b).

Data used for the contrast variation experiments were also collected on EQ-SANS using only the longer sample to detector distance to achieve a coverage from 0.009 Å ^−1^ *< q <* 0.06 Å ^−1^. The data were evaluated as the Porod invariant, Q*, which is readily compared to the total scattering intensity I (0). Collection times for these observations were approximately 20 min for each solvent contrast condition.

## Results and discussion

### Growth characteristics during CRISPRi induction

**FIGURE 1:**
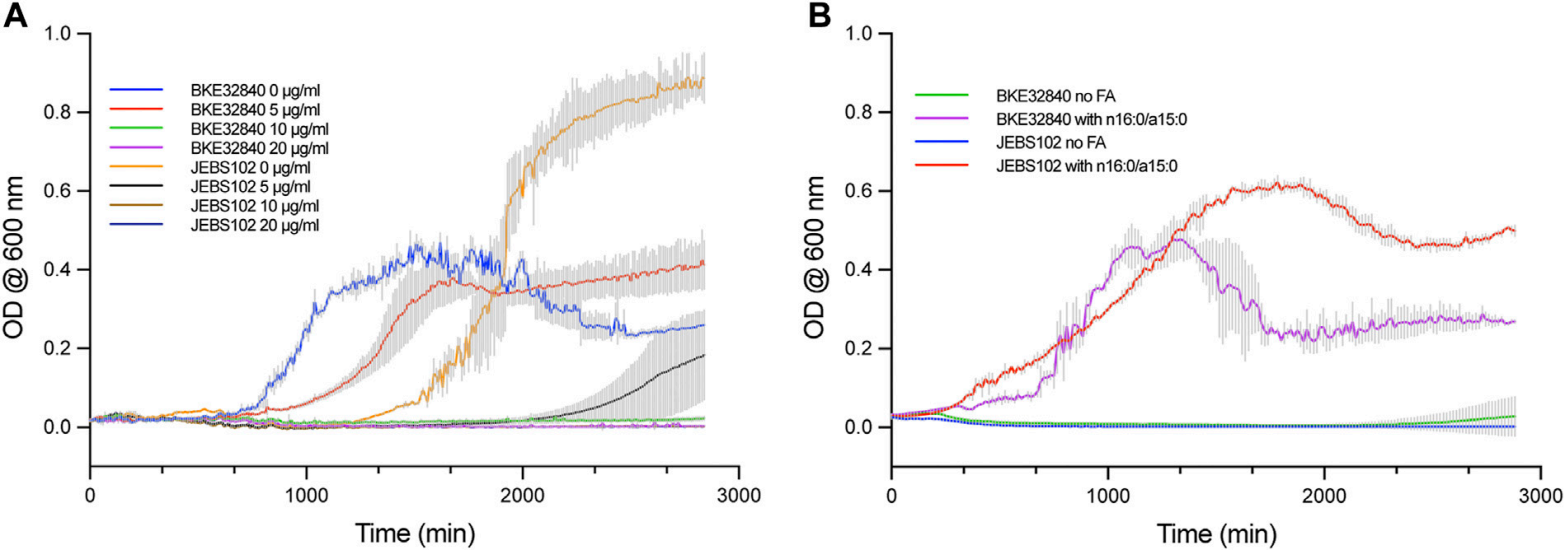
Comparative growth assays were conducted in 96-well format between *B. subtilis* strain JEBS102 (*Em* ^*r*^ *Cm* ^*r*^ *trpC2* Δ*fadN lacA::P*_*xyl*_ *-dcas9 amyE::P*_*veg*_ *-sgRNA* (*fabF*)), and the original strain BKE32840 (*trpC2* Δ*fadN::erm*). **(A)** Cell growth was compared between the two strains in M9-Gluc, 5 mM L-tryptophan, 1% (*w/v*) D-xylose, plus the addition of 0, 5, 10, and 20 μg/ml of cerulenin. In the absence of cerulenin, BKE32840 displayed a shorter lag time but ca. 2-Fold lower cell densities that JEBS102. A cerulenin concentration of 5 μg/ml did not inhibit growth of BKE32840 but significantly inhibited JEBS102 with low turbidity developing in some wells after 37 h of incubation. Higher concentrations of cerulenin blocked growth of both strains. **(B)** *B. subtilis* strains JEBS102 and BKE32840 were grown in M9-Gluc (with 5 mM L-tryptophan), 1% (*w/v*) D-xylose, 20 μg/ml cerulenin, 5 g/L FA-free BSA and either with or without the addition of 8 μg/ml each of *n*16:0 and *a*15:0. The addition of FAs rescued the growth of both strains with JEBS102 displaying a slower rate of growth but higher overall cell densities. No growth occurred in the absence of exogenous FAs. Each data point represents the mean of three biological replicates with error reported as ± standard deviation.

Our previously described system to introduce specific FAs into the plasma membrane of *B. subtilis* BKE32840 growing in a high D_2_O background enabled novel experimental approaches to study both transverse and lateral membrane structure in living bacteria using SANS techniques (Nickels et al., 2017b). However, as *fabF* is an essential gene in *B. subtilis* (Peters et al., 2016), arresting *de novo* fatty acid synthesis relied solely on the activity of 50 μg/ml cerulenin to inhibit incorporation of unwanted FAs into the membrane. In this work, we have leveraged the CRISPRi knockdown library constructed by Peters et al. (2016) for inhibiting transcription of essential genes in *B. subtilis* Essential Genes to generate a new strain, *B. subtilis* JEBS102, and compared its sensitivity to cerulenin against BKE32840. In cultures growing in M9-Gluc without the addition of exogenous FAs, the JEBS102 strain displayed noticeably different growth kinetics versus BKE32840, especially at lower doses of cerulenin (Figure 1A). JEBS102 exhibited a longer lag time (approximately 10 h) than BKE32840 in the absence of cerulenin likely due to the induction of dCas9 by the addition of D-xylose (Figure 1A). However, we have consistently observed that JEBS102 achieves 1.5–2.0 fold higher overall cell densities and we are currently investigating possible explanations for this result. In the presence of 5 μg/ml of cerulenin, BKE32840 was only slightly inhibited in growth rate vs the no cerulenin control while JEBS102 showed variable break-out after ∼37 h of incubation, though some wells produce no turbidity indicating complete inhibition. At higher concentrations of cerulenin, no growth was observed in either strain.

JEBS102 responded consistently well when the binary mixture of *n*16:0 and *a*15:0 FAs were exogenously supplied to cultures while under D-xylose induction with 20 μg/ml of cerulenin (Figure 1B). BKE32840 displayed variable growth with an increased lag time and a sharp decrease in cell density after ∼22 h. The JEBS102 strain produced significantly higher cell densities, albeit at a slower growth rate than BKE32840 but with a significantly reduced death phase which greatly facilitates FA-fed cell sample preparation for samples directed for SANS experiments which require 10 mg/ml cell dry weight for adequate data collection while under the neutron beamline.

### Fatty acid uptake and neutron contrast

**FIGURE 2:**
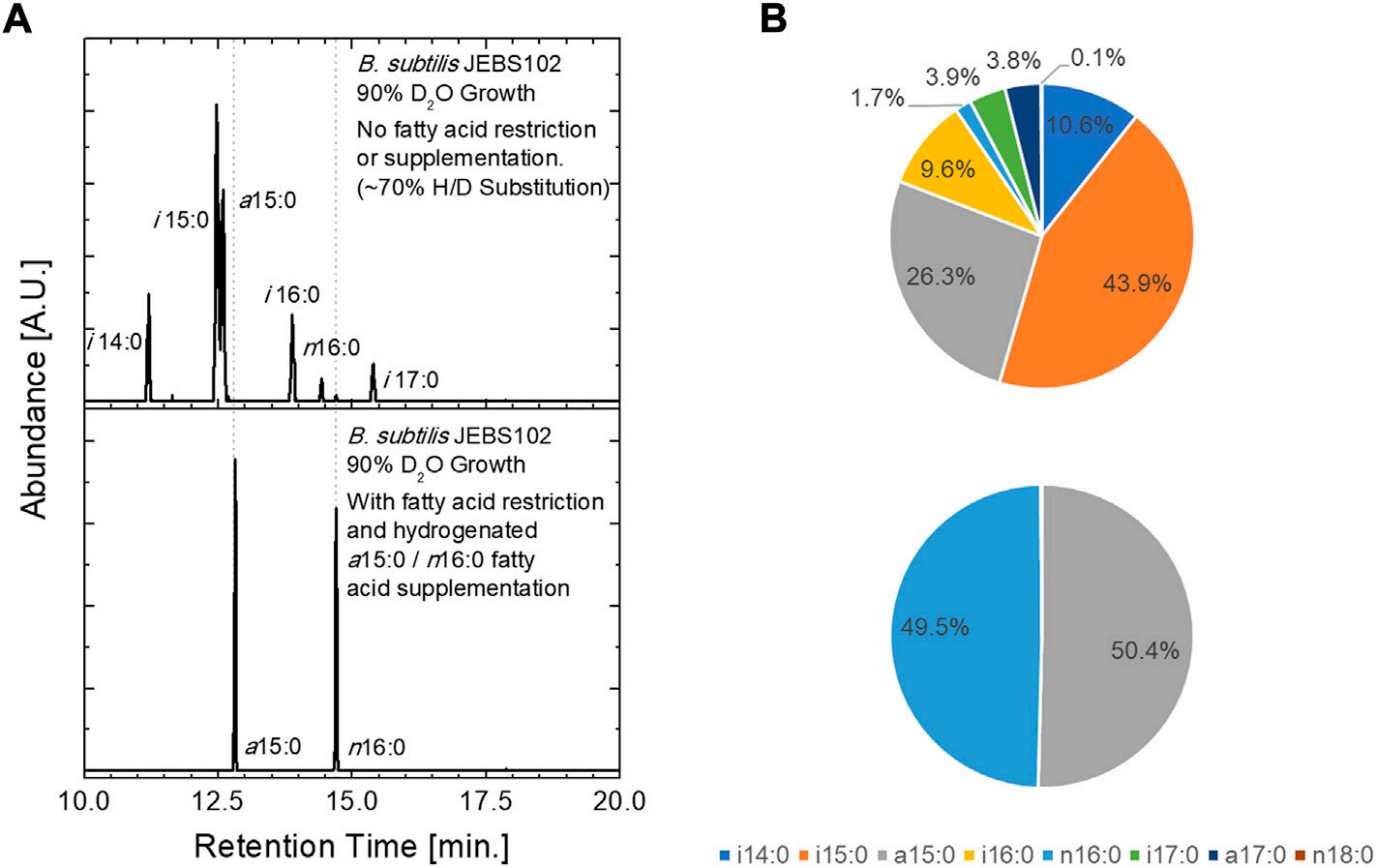
Fatty acid uptake was assessed using GC/MS analysis of extracted FAs from *B. subtilis* JEBS102 cells grown in 90% D_2_O growth conditions, with or without FA restriction and supplementation using the procedures described. **(A)** Chromatograms illustrate the presence of the seven native FAs in the upper panel and only the two supplemented FAs in the lower panel. Deuterium inclusion in synthesized FAs is indicated by both the decreased elution time and the broadening of the FAME peaks. The trace amounts of *n*18:0 and *n*16:0 are contaminants from release agents on plastic labware. **(B)** Pie charts summarize the FA uptake described in **(A)**. Notable differences in FA uptake between the current construct and prior work can be seen, namely a different ratio of *i*15:0 to *a*15:0 in the ‘native’ mixture of FAs in the uninduced cells and a far greater ratio of *n*16:0 to *a*15:0 in the induced and FA rescued cells.

The incoproation of isotopically labeled FAs was assessed *via* GC/MS and by contrast variation SANS. In these experiments, *B. subtilis* JEBS102 cells were grown in media prepared with 90% D_2_O containing media. This results in ∼70% deuterium substitution in the carbon backbone of the biomolecules within the cell (as determined *via* GC/MS) and generates a minimum neutron scattering condition when the cells are suspended in an 85% D_2_O containing buffer (Figure 2). This is desirable, as minimizing total scattering of the cells at high fractions of D_2_O minimizing incoherent background scattering. Natural abundance hydrogen FAs can be introduced upon xylose induction and incorporated into the cellular membranes of these partially deuterated cells by supplementing the culture medium with H-labeled FAs. GC/MS results obtained from unlabeled cells cultured in 90% D_2_O exhibit the expected seven FAMEs (Kaneda, 1977) from cells (upper panel). The partially deuterated FAMEs elute earlier than their natural abundance hydrogen equivalent, and their associated peaks are broader due to the presence of a distribution of isotopomers. In the lower panel, we observe that cells rescued by the addition of two FAs, H-anteiso-pentadecanoic acid (*a*15:0) and H-normal-hexadecanoic acid, contain only the two supplemented H-FAs, and no deuteration as seen in the unlabeled cells, proving the update and incorporation of supplemented FAs.

Interestingly, a higher ratio of *n*16:0 to *a*15:0 FAs (approx. 1:1) was incorporated in the cell membrane of the labeled *B. subtilis* JEBS102 than in the prior approach (Nickels et al., 2017b) which depended only on cerulenin and had a nearly 1:5 ratio of *n*16:0 to *a*15:0. Note that the exogenous FAs are added in a 1:1 ratio. We do not offer a mechanistic explanation for this observation, as subsequent study is required. We can, however, speculate that inhibition of FA synthesis at the transcription level impacts regulation (Fujita et al., 2007) of membrane synthesis and fluidity differently than cerulenin alone. Increased content of *a*15:0 FA would have a fluidizing effect on the cell membrane, relative to the current 1:1 ratio. The reported response of *B. subtilis* to cerulenin is a significant increase in FabF content (Schujman et al., 2001; Nickels et al., 2020), due to the accumulation of malonyl-CoA which disrupts the *fapR* repressor-operator complex (Schujman et al., 2003; Schujman et al., 2006; Fujita et al., 2007; Albanesi and De Mendoza, 2016). Interactions at the transcript level using the current CRISPRi approach need to be interrogated further. It is also possible that the change in FA content is somehow connected to the initial uptake of exogenous FAs through the FakAB system.

**FIGURE 3:**
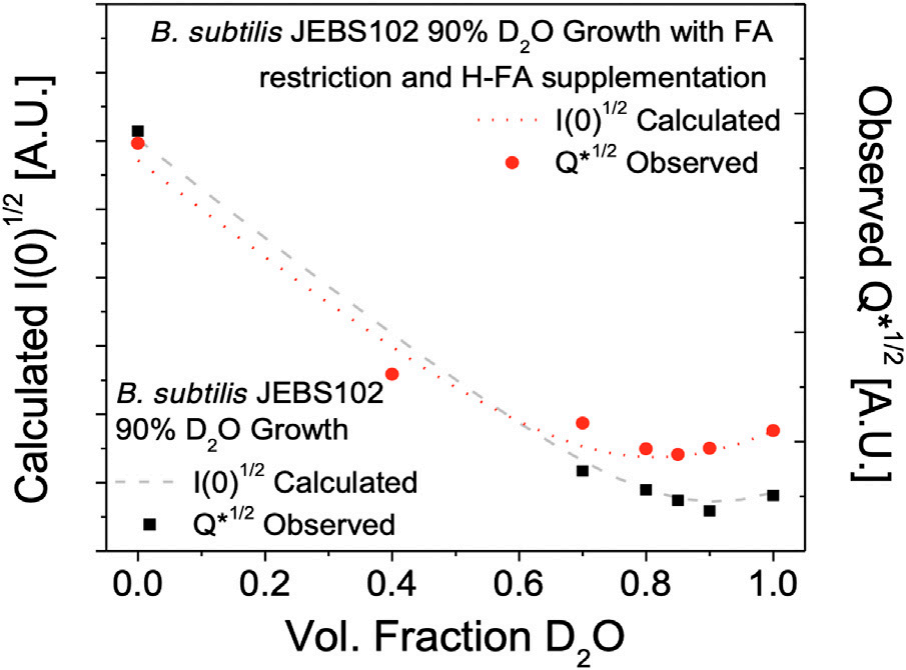
Total neutron scattering from *B. subtilis* JEBS102 cells grown in 90% D_2_O growth conditions, with or without FA restriction and supplementation using the procedures described as a function of D_2_O content in the resuspension buffer. Total scattering was assessed as the Porod invariant, Q*, over the range from 0.009 Å −1 < *q* < 0.06 Å −1. This is compared to the estimated total scattering intensity *I(0)* based on a model (Nickels et al., 2017b) of estimated biomolecule content and scattering length density. The incorporation of hydrogenated FAs in the partially deuterated cells leads to the observed increase in scattered intensity of FA labeled cells at high fractions of D_2_O in the resuspension buffer.

Contrast variation SANS experiments were performed on the same partially deuterated versions of H-fatty acid labeled and unlabeled *B. subtilis* JEBS102 cells - resuspended in varying ratios of H_2_O to D_2_O containing resuspension media. The resulting scattering was evaluated as the Porod invariant (Figure 3):

$$Q^{*}=\int q^{2}I\left(q\right)dq\propto \frac{2\pi I\left(0\right)}{V_{p}}$$ (1)

where *q* is the scattering wave vector, and *I*(*q*) is the observed scattering for a given solvent contrast. This quantity is also proportional to the forward scattering, *I* (0), which is useful quantity since *I(0)* can be modeled from the cellular compositions and deuteration level as described below. V_p_ is the volume of the scattering particle. The observed scattered intensity Q* is shown along with the calculated intensities *I* (0) which shows a global minimum of scattering in the unlabeled cells at approximately 85% D_2_O in the resuspension buffer. This is in close agreement with prior work (Nickels et al., 2017b). Similarly, a significant increase in scattering in observed in the H-fatty acid labeled cells due to the strong scattering of the hydrogenated FAs in the cell membrane of the labeled cells, confirming the incorporation of the natural abundance hydrogen FAs and identifying 85% D_2_O resuspension buffer as the target condition for detailed structural measurements of the labeled cell membrane.

The total forward scattering, *I* (0), from the labeled and unlabeled cells was estimated using the model put forward in Nickels et al. (Nickels et al., 2017b). Scattering arises from the square of the difference in scattering length density, *ρ*, between a molecular species (*m*) and the surrounding medium (*s*), ${\left(\rho_{m}-\rho_{s}\right)}^{2}$ . For a whole cell, we can consider the total forward scattering to be proportional to the sum of this difference squared for each class of biomolecules (*i*), weighed by its relative volume fraction, *χ_i_*, as:

$$I\left(0\right)\propto \overset{n}{\underset{i=1}{\sum }}\chi_{i}{\left(\rho_{i}-\rho_{s}\right)}^{2}$$ (2)

To model *ρ* for each class of biomolecule we can apply the relationship:

$$\rho =\rho_{H}+f_{XD}\Delta \rho_{XD}+f_{CD}\Delta \rho_{CD}$$ (3)

where *ρH* is the natural abundance hydrogen scattering length density for a given biomolecule class, *f*_*XD*_ and *f*_*CD*_ are the fractions of deuterium substitution for hydrogens bound to heteroatoms (*X* = *N*, *O*, *S*) or bound to carbon, Δ*ρ*_*XD*_ and Δ*ρ*_*CD*_ are represent the increase in ρ from complete deuteration of the given biomolecule class.

Table 1 describes the input values used. The overall dry composition data was taken from Bishop (Bishop et al., 1967) with an assumed water content of 80% by weight. *B. subtilis* dry mass is made up of protein (53%), RNA (18%), DNA (2.6%), lipid (5.2%), and carbohydrate (2.8%). The remainder consists of small organic molecules, such as amino acids, cofactors, and nucleotides plus inorganic material. All neutron scattering lengths are from Sears (1992) and were obtained through the NIST Center for Neutron Research (https://www.ncnr.nist.gov/resources/n-lengths/). The SLD values for protein were based on residue volumes for amino acids taken from Zamyatnin (1984). RNA content was taken from Midgley (1962), and residue volumes were from Voss and Gerstein (2005). The G + C content of *B. subtilis* 168 DNA (43.5%) was used here and taken from the genomic sequence (Kunst et al., 1997), with residue volumes fromNadassy et al. (2001). The SLD of the carbohydrate composition (CHO) was estimated from chitin, i.e., poly (*N*-acetylglucosamine) with an experimental density of 1.425 as reported by Li et al. (1996). The CD substitution for lipids was found to be 69% from GC/MS consistent with the expected deuteration from prior work on *B. subtilis* (Nickels et al., 2017b). The protein deuteration was taken to be 60% based on prior work (Leiting et al., 1998; Nickels et al., 2017b). Other biomolecules were assumed to have 70% CD substitution and water-exchangeable positions was assumed to match that of the medium. Volume percentages (reported for natural abundance hydrogen species) are assumed to be invariant with respect to deuteration, though mass percentages and density would vary to a small degree. A detailed breakdown of the model for each biomolecule class in *B. subtilis* can be found in our earlier work (Nickels et al., 2017b).

**TABLE 1:** Abundances and selected physical properties of major cellular species.

Species	Mass %	Density (g/cm^3^)	Volume %	*ρ*_*H*_ (fm/Å^3^)	Δ*ρ*_*XD*_ (fm/Å^3^)	Δ*ρ*_*CD*_ (fm/Å^3^)
Protein	13.0	1.39	9.9	0.19	0.14	0.47
RNA	4.4	1.76	2.7	0.35	0.11	0.26
DNA	0.6	1.67	0.4	0.32	0.07	0.32
CHO	0.7	1.43	0.5	0.18	0.13	0.44
Lipid	1.3	0.98	1.4	–0.035	0.00	0.67
Water	80.0	1.00	85.1	–0.056	0.70	0.00

### Cell membrane structure

**FIGURE 4:**
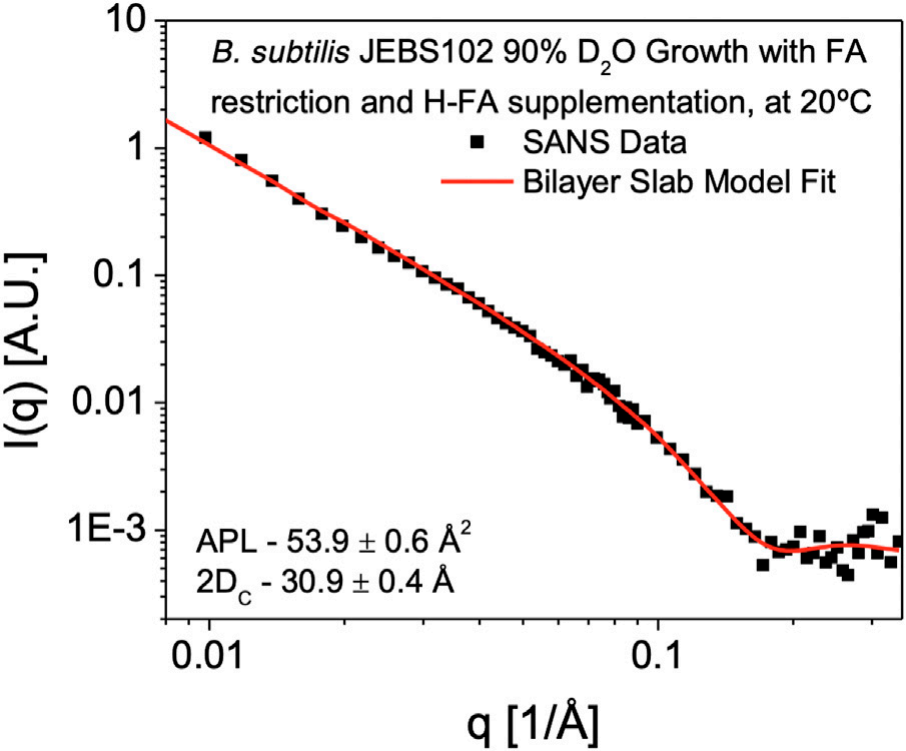
SANS Measurement of Cell Membrane hydrophobic Thickness. SANS data obtained from *B. subtilis* JEBS102 cells grown in 90% D_2_O media, induced to block expression of *fabF* and rescued with supplementary FAs, H-anteiso-pentadecanoic acid (*a*15:0) and H-normal-hexadecanoic acid (*n*16:0) in 85% D_2_O resuspension media is plotted as scattered intensity, *I(q)*, as a function of scattering wavevector, q (Å^−1^). Error bars correspond to ± σ. The experimental data are shown superimposed with the best-fit (red solid line) using a self-consistent lamellar form factor (Tan et al., 2021) and the parameters described in Table 1, 2 revealing an average hydrophobic thickness of 30.9 ± 0.4 Å. The form factor is consistent with contrast being introduced exclusively within the hydrophobic portion of the cell membrane.

SANS measurements to determine membrane hydrophobic thickness were performed on H-FA labeled cells in 85% D_2_O resuspension buffer. Subtraction of the background scattering from the sample scattering reveals a scattering curve (*I(q)* versus *q* data) characteristic of a lamellar structure (Figure 4). This is characterized by a *q* ^−2^ dependence at low-*q*, arising from the 2D membrane surface of the entire bacterium, transitioning to a *q* ^−4^ dependence typical of 3D objects with smooth surfaces and sharp interfaces. Formally this can be modeled using a lamellar form factor representing the acyl core of the bilayer, with two symmetrical and identical head group regions on either side (Tan et al., 2021), which at 85% D_2_O are assumed to be contrast-matched to the scattering density of the surrounding cellular environment. Using the self-consistent lamellar model, we obtain an estimate of the area per lipid (APL) of 53.9 ± 0.6 Å^2^. Using the GC/MS measurements and model of the bilayer composition summarized in Table 2, we can convert this into an estimate of the average membrane hydrophobic thickness (2*D*_*C*_) as 30.9 ± 0.4Å at 25°C.

**TABLE 2:** Input parameters for the FA core of lamellar model (Tan et al., 2021) of cell membrane structure taken from GC/MS measurements and the *B. subtilis* lipid extract model (Nickels et al., 2017a).

Fatty acid	Mole (%)	Vol. (Å3)	Vol (%)	*B* (fm)	*ρ* (fm/Å3)	MW (g/Mole)
*i*14:0	0.0	384.3	0.0	-22.1	-0.05	197
*i*15:0	0.0	412.2	0.0	-22.9	-0.05	211
*a*15:0	50.4	412.2	49.9	-22.9	-0.05	211
*i*16:0	0.0	440.1	0.0	-23.8	-0.05	225
*n*16:0	49.5	420	50.0	-23.8	-0.05	225
*i*17:0	0.0	468	0.0	-23.6	-0.05	239
*a*17:0	0.0	468	0.0	-23.6	-0.05	239
*n*18:0	0.1	475.8	0.1	-23.4	-0.05	253
Average per chain		416.1		-23.3	-0.05	221
Average per 2-chain lipid		832.2		-46.7	-0.05	441.9

Utilizing the prior approach (Nickels et al., 2017b), the hydrophobic portion of the bilayer, *2D*_*C*_, was observed to be somewhat thinner (24.3 ± 0.9Å at 25°C) than we observe here. This is unsurprising given the observed compositional difference, with the prior approach yielding labeled cells containing greater than 80% of the low melting FA *a*15:0 and less than 20% of the higher melting component, *n*16:0. This is compared to the 1:1 ratio of *n*16:0 to *a*15:0 FAs we see in this study. The increased *n*16:0 content would likely lead to a thicker and more ordered cell membrane. It would be a reasonable expectation that this will lead to changes in lipid head group distribution to regulate membrane fluidity. The *B. subtilis* 168 strain lipid extract model (Nickels et al., 2017a), with a more diverse seven FA composition was also found to have a thinner hydrophobic core on the order of 25 Å at 25°C. Yet we are still in the range of hydrophobic thickness for the phosphatidylcholine (PC) bilayers, such as dimyristoyl PC (2*D*_*C*_ = 25.7 Å at 30°C) or 1-palmitoyl-2-oleoyl PC (2*D*_*C*_ = 28.8 Å at 30°C) (Kučerka et al., 2011) or dipalmitoyl PC (2*D*_*C*_ = 29.4 Å at 50°C) (Fujita et al., 2007).

Despite the contrast strategy preventing a direct observation of the headgroup region (i.e., the headgroup region matches the solvent and cellular background), an estimate of the dimensions of the full cell membrane can still be offered based on assumptions of the water content and head group composition of the *B. subtilis* 168 strain lipid extract model (Nickels et al., 2017a). These approximations will likely vary from the reality given our expectation of head group compositional changes in response to the FA content and the labeling process in general (Nickels et al., 2020). Nevertheless, we can calculate the headgroup thickness by assuming an average per lipid headgroup volume, *V*_*H*_ from Table 3, and water content, *n*_*W*_. The self-consistent lamellar model provides a value for APL which we can use *via*:

$$D_{H}=\frac{V_{H}+n_{W}V_{W}}{APL}$$ (4)

where *V*_*W*_ is the water molecular volume. Taking *n*_*W*_ to be 11.6, *V*_*W*_ to be 30.4 Å^3^, *APL* to be 53.9 Å^2^, and *V*_*H*_ to be 258.9 Å^3^; we obtain an estimate of 11.3 ± 0.2 Å for *D*_*H*_.

**TABLE 3:** Input parameters for the headgroup portion of lamellar model (Tan et al., 2021) of cell membrane structure taken from the *B. subtilis* lipid extract model (Nickels et al., 2017a). This portion of the membrane is contrast matched to 85% D_2_O resuspension buffer, making the resulting fit insensitive to this part of the membrane structure but if water content equal to the extract is assumed, n_W_ = 11.6, a headgroup thickness (D_H_) of 11.3 ± 0.2 Å can be inferred.

Headgroups	Mole (%)	Vol. (Å^3^)	Vol (%)	*B* (fm)	*ρ* (fm/Å^3^)	MW (g/Mole)
PE	54.2	317.8	66.3	185.93	0.59	269
PG	9.3	325.1	11.6	182.87	0.56	299
CL	3.2	498.4	6.1	264.05	0.53	505
DAG	31.3	151.8	18.3	88.20	0.58	146
FFA	2.0	44.1	0.3	24.90	0.56	28
Average per 2-chain lipid		258.9		150.6	0.58	228.5

PE, phosphoethanolamine; PG, phosphoglycerol; CL, cardiolipin; DAG, diacylglycerol; FFA, free fatty acid.

## Conclusion

Selective introduction of protonated or deuterated FAs into growing cells of *B. subtilis* have previously relied exclusively on the use of the expensive and unstable fungal-derived small molecule cerulenin. Here, we employed CRISPRi tools to modulate fatty acid biosynthesis and provide an improved strategy for selectively incorporating protonated or deuterated FAs into growing cells of *B. subtilis* by inhibiting *fabF* expression in a xylose-dependent manner. The resulting strain (JEBS102) showed greater sensitivity to cerulenin and produced 1.5–2.0 fold higher overall cell densities that greatly facilitated more robust sample preparations. This procedure was compatible with deuterated growth conditions which enables SANS experiments to determine transverse membrane properties in viable cells. The new strain also demonstrated precise uptake of two exogenously supplied FAs to construct a functional plasma membrane. A difference in FA uptake ratio was observed relative to the earlier procedure, with a relative increase in the amount of incorporated *n*16:0 FAs. This change manifested in a larger membrane thickness observed *via* SANS. Differential isotopic labeling of cell membrane systems *in vivo* is an enabling methodology for a new generation of structural studies in living systems. Beyond neutron scattering, vibrational spectroscopy and NMR methods are sensitive to isotopic labels such as deuterium. This implies that this labeling approach will be of interest to a range of membrane researchers, utilizing other methods.

## Data Availability

The original contributions presented in the study are included in the article/Supplementary Material, further inquiries can be directed to the corresponding author.
