# Supplementary material for: Improved chemical and isotopic labeling of biomembranes in Bacillus subtilis by leveraging CRISPRi inhibition of beta-ketoacyl-ACP synthase (fabF)
Source: Front Mol Biosci. 2022 Oct 21;9:1011981. doi: 10.3389/fmolb.2022.1011981 (PMC9634059; doi:10.3389/fmolb.2022.1011981)
Supplement: Supplementary file 1 [file DataSheet1.docx]

**Supplementary Table S1**. Strain designations, microorganisms, genotypes and source of strains with references used in this study.

| **Strain** | **Organism** | **Genotype** | **Source** | **Reference** |
| --- | --- | --- | --- | --- |
| BKE32840 | *Bacillus subtilis* subsp. *subtilis* 168 | Em^r^ *trpC2* Δ*fadN* | BGSC | Koo et al., 2017 |
| 1A1278 | *Bacillus subtilis* subsp. *subtilis* 168 | Em^r^ *trpC2* *lacA*::P*_xyl_*-dcas9 | BGSC | Peters et al., 2016 |
| BEC11340/  CAG74640 | *Bacillus subtilis* subsp. *subtilis* 168 | Cm^r^ Em^r^ *trpC2 lacA*::P*_xyl_*-dcas9 *amyE*::P*_veg_*-sgRNA(*fabF*) | BGSC | Peters et al., 2016 |
| ECE274 | *Escherichia coli* | pDR244 Ap^r^ Sp^r^ | BGSC | Koo et al., 2017 |
| JEBS100 | *Bacillus subtilis* subsp. *subtilis* 168 | Em^s^ *trpC2* Δ*fadN* Δ*erm* |  | This work |
| JEBS101 | *Bacillus subtilis* subsp. *subtilis* 168 | Em^r^ *trpC2* Δ*fadN lacA*::P*_xyl_*-dcas9 |  | This work |
| JEBS102 | *Bacillus subtilis* subsp. *subtilis* 168 | Cm^r^ Em^r^ *trpC2* Δ*fadN lacA*::P*_xyl_*-dcas9 *amyE*::P*_veg_*-sgRNA(*fabF*) |  | This work |

BGSC, Bacillus Genetic Stock Center

**Supplementary Table S2.** Primers used in this study for PCR verification of strain construction.

| **Name** | **Sequence (5’-3’)** | **Binding Site** |
| --- | --- | --- |
| BSU32840-F | AGGCGAGTATGCAGCTTCG | 5’ region of *fadN* |
| BSU32840-R | TCCGGCAAGCTTCAAAGC | 3’ region of *fadN* |
| 854-F | GTCATGGGGGCCAGCTATAC | 5’ region of *lacA* |
| 862-R | GAGGTCCCTAGACTCTAGACCC | pAX01 vector backbone |
| 3200-F | GGGTTTTTCTGGGGAAATGGG | pAX01 vector backbone |
| 3544-R | ACCTTCATTCCGTTCGCTGT | 3’ region of *lacA* |
| amyE-F | ATGTTTGCAAAACGATTCAAAACCT | 5’ region of *amyE* |
| amyE-R | TCAATGGGGAAGAGAACCGC | 3’ region of *amyE* |
| 1588-F | GTCAGATAGGCCTAATGACTGGC | 3’ region of *cat* (Cm^r^) |
| 2562-R | CCACAAGCTCATCTGTGATTCC | 3’ region of *amyE* |

**References:**

Koo, B.-M., Kritikos, G., Farelli, J.D., Todor, H., Tong, K., Kimsey, H., Wapinski, I., Galardini, M., Cabal, A., and Peters, J.M.J.C.S. (2017). Construction and analysis of two genome-scale deletion libraries for *Bacillus subtilis*. *Cell Syst*. 4**,** 291-305. e297.

Peters, J.M., Colavin, A., Shi, H., Czarny, T.L., Larson, M.H., Wong, S., Hawkins, J.S., Lu, C.H., Koo, B.M., Marta, E. and Shiver, A.L., 2016. A comprehensive, CRISPR-based functional analysis of essential genes in bacteria. *Cell*, 165(6), pp.1493-1506.
